# A Deep Classifier for Upper-Limbs Motor Anticipation Tasks in an Online BCI Setting

**DOI:** 10.3390/bioengineering8020021

**Published:** 2021-02-05

**Authors:** Andrea Valenti, Michele Barsotti, Davide Bacciu, Luca Ascari

**Affiliations:** 1 Department of Computer Science, University of Pisa, 56127 Pisa, Italy; bacciu@di.unipi.it; 2 CAMLIN Italy s.r.l., 43121 Parma, Italy; m.barsotti@camlintechnologies.com (M.B.); l.ascari@camlintechnologies.com (L.A.)

**Keywords:** deep learning, brain–computer interfaces, artificial neural networks

## Abstract

Decoding motor intentions from non-invasive brain activity monitoring is one of the most challenging aspects in the Brain Computer Interface (BCI) field. This is especially true in online settings, where classification must be performed in real-time, contextually with the user’s movements. In this work, we use a topology-preserving input representation, which is fed to a novel combination of 3D-convolutional and recurrent deep neural networks, capable of performing multi-class continual classification of subjects’ movement intentions. Our model is able to achieve a higher accuracy than a related state-of-the-art model from literature, despite being trained in a much more restrictive setting and using only a simple form of input signal preprocessing. The results suggest that deep learning models are well suited for deployment in challenging real-time BCI applications such as movement intention recognition.

## 1. Introduction

Decoding motor intentions from non-invasive brain activity monitoring (e.g., electroencephalogram, EEG) is one of the most challenging aspects in the Brain Computer Interface (BCI) field. Indeed, the possibility to reliably detect the planning of movements, and thus to anticipate the motor action, would positive affects the usage of both restorative and assistive BCI systems for rehabilitation of motor paralysis and to improve life quality of people for which brain-based communication would be the only viable option to interact. There is now ample evidence that non-invasive restorative BCI can have a benefit relative to conventional neuro-rehabilitation approaches in patients with serious motor disability [1,2,3]. However, the early detection of movement intention remains an open challenge for enhancing neuro-feedback interventions, in which the precise feedback timing is essential for promoting neuro-plasticity by contingently closing the loop between the motor information (patient’s volitional effort) and the sensory information (motor feedback often provided by FES or robotic exoskeletons). Regarding the assistive BCI, the detection of motor planning could pave the way for the establishment of intimate and natural interaction with external devices such as human-support robots, orthosis, wheelchair, etc., for those individual presenting highly impeded motor capabilities [4]. Indeed, together with the rapid scientific and technological growing in the fields of human sensing and Artificial Intelligence (AI), we are witnessing a growing interest of the industry production in BCI devices, which could enable the “natural” operation of external devices for both healthy subjects and especially for those subjects that would benefit more from the usage of BCI systems [5].

EEG-based BCI systems aimed at capturing motor-correlates, rely mostly on the processing of SMRs (sensory motor rhythms) [6] or MRCPs (movement related cortical potentials) [7]. The SMRs processing refers to the detection of event-related desynchronization/synchronization (ERD/ERS) [8] that are modulations of rhythmic activities (mu, beta, and gamma rhythms) recorded over the sensorimotor cortex, whereas MRCPs are slow EEG variations related to both planning and execution of both imagined and executed movements [9,10]. Generally, supervised and extensive processing steps are required for the extraction and analysis of both SMR and MRCP, usually aimed at finding spatial and frequency filters that maximisez the feature separability among desired movement classes. In the detection of the motor-related band-power modulations (SMR), the optimization of spatial filters is generally performed with the application of the common-spatial pattern (CSP) that can be combined with frequency domain information extracted through filter banks (FB-CSP) [11], or through the wavelet transform decomposition [12]. Analogously, regarding the time-domain amplitude modulations (MRCP), the detection is usually performed through the application of an optimised spatial filter (performed by several techniques ranging from discriminative spatial patterns DSP [13,14] to constrained ICA [15]) combined with frequency filters focused in the delta rhythm. After the features extraction process, for both the SMR and MRCP processing, a classification stage is required for discriminating the desired movement class, and it is usually performed with standard machine learning techniques (e.g., LDA, SVM, random forest). As it is well known, due to the high non-stationarity nature of EEG signals, the extraction of the above mentioned movement-associated features is a difficult and time consuming task, also requiring an extensive prior knowledge of the neural processes behind movements.

For this reason, and thanks to the rapid advancements in the AI field [16], an increasing number of works focuses on the application of deep learning (DL) methods for the recognition of mental states associated with movements from EEG signals [17] and also from the combination of EEG and electromyiographic (EMG) signals [18]. In a recent work [19], it has been demonstrated that three different DL models (namely long short-term memory, LSTM, Recurrent Neural Network, RNN, and Convolutional Neural Networks, CNN) obtained better overall performance in decoding motor imagery movements when compared to state-of-the-art machine learning techniques. This result came with the double-fold advantage of avoiding any feature engineering step while easily translating offline results in a real-time application. However, motor-imagery strategy, although being a valid substitute for active motor training as a mean to activate the motor network [20], it is not suitable for BCI-applications aimed at controlling external avatar/robots because it suffers from a delay between intention and control command. This would result in an unnatural control strategy.

Indeed, several recent works are focusing on the detection of motor anticipation rather than the detection of motor-imagery/execution, with envisaged applications ranging from driving [21] to the control of robotic devices [22,23]. The application of DL techniques for classifying motor planning of different movements has been recently investigated by the important work of Mammone et al. [24], providing also an updated overview of relevant works about EEG-based motor anticipation. In their work, they proposed to classify 6 different movement classes, plus the rest class, by using only a sub-part of the EEG signal corresponding to the second before the movements onset. A set of 21 binary classifiers were implemented and each classification has been performed by feeding a CNN with 1 s of temporal evolution data of 43 Frequency bins maps (extracted with Continous Wavelet Transfer in the 0.6–45 Hz range) related to 210 back-projected reconstructed sources. The source reconstruction inverse problem is solved using the beamforming method and only sources related to supplementary motor and primary motor are retained. Despite the goodness of their results, and the interesting physiological interpretation of their findings, it has to be considered that only the accuracy of each single binary classifier is reported without considering strategies for their combination. Moreover, their approach is not suitable for being easily translated in a real-time application, due to the computational effort required by the the source reconstruction process and time frequency decomposition steps.

### Contributions of This Work

With the aim of overcoming the limitations of “handmade” feature extractions and signal processing, and to provide a practical alternative to the panel of binary classifiers, in this work we propose a DL-based architecture for movement anticipation detection. The model takes in input the raw EEG data, which has been spatially re-organised in a topology-preserving representation that is able to keep the information about the spatio-temporal dependencies of the channels. We tested our approach using the same publicly available dataset used by Mammone et al. [24], consisting in a high density EEG recording acquired simultaneously with motion data (described in detail in Ofner et al. [14]). The major contributions of our work can are the following:

- We introduce a new input representation of EEG data that allows to preserve the spatio-temporal dependencies between the different channels.
- We develop a novel convolutional deep learning model for the efficient processing of raw EEG data.
- We compare our model with previous results on the same dataset, showing that our approach leads to a significantly higher accuracy with the advantage of a sensibly reduced processing overhead.

Showing that deep learning architectures can be successfully applied on EEG-based motor anticipation tasks, this work paves the way for the use of deep learning models in challenging online BCI settings.

The rest of the paper is structured as follows: in Section 2, we present in detail the methodology used in this work. Section 2.1 introduces the dataset on which the model has been evaluated, with Section 2.2 listing the data epoching and the movement onset detection heuristics employed. Section 2.3 describes the signal preprocessing techniques that have been used, as well as presenting the novel 3D spatio-temporal representation of the inputs. Section 2.4 introduces the detailed architecture of the model, specifically focusing on the Encoder component in Section 2.4.1 and on the Classifier component in Section 2.4.2. The training scheme of the model is reported in Section 2.5, while in Section 2.6 we describe how the results presented by [24] have been adapted in order to allow a direct comparison with our results. Section 3 presents the empirical analysis, first by reporting the classification accuracy on the selected epoch for the different subjects (Section 3.1), then by showing the accuracy of the classifier over time around the movement onset (Section 3.2). In Section 4, we discuss the results, especially in the context of a possible application of the model in an online setting. Finally, we outline some possible research directions that we are willing to explore in the future.

## 2. Methods

### 2.1. Dataset

**Figure 1:**
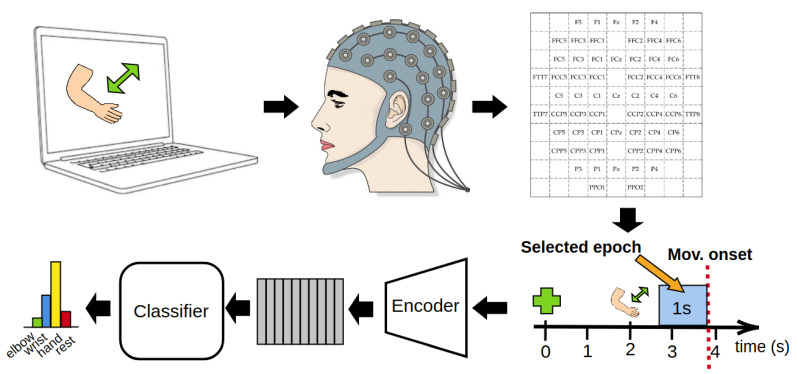
Conceptual schema of the dataset and the acquisition protocol used in this work. The acquired raw electroencephalogram (EEG) data is first arranged in our dependendancy-preserving representation. Then, we keep the 1-second segment preceding the movement onset as our training epoch. Each training segment is then encoded and fed to our model to get the final classification.

In order to encourage reproducibility, we chose to use a public dataset, available at the BNCI Horizon 2020 website (http://bnci-horizon-2020.eu/database/data-sets, accessed on 22 December 2020). In Figure 1 we report a conceptual schema of the dataset and the acquisition protocol used. This dataset was chosen because it is the only publicly available BCI dataset of high-density EEG signal synchronized with motion data, recorded via an exoskeleton and a glove [14]. Such motion data, while not used for the training of the model, is crucial for determining the exact start of the movement. For an in-depth description of the dataset, we refer the interested reader to [14]. The EEG data is coming from 61 electrodes distributed over the frontal, central, parietal and temporal areas of the skull. The raw data is collected for both motor imagery and motor execution tasks from 15 healthy subject, 6 males and 9 females, aged from 22 to 40 years, with a mean age of 27 ± 5 years. Subjects, with their right arm connected to an anti-gravity exoskeleton (in order to avoid muscle fatigue), sit on a chair in front of a computer screen, positioning their hand in the starting neutral position (hand half open, lower arm extended to 120°, neutral rotation). The subjects are required to perform six types of actions (elbow flexion, elbow extension, wrist pronation, wrist supination, hand open, hand close), plus an additional “rest action”, for a total of 7 different movement classes. Ten runs of data acquisitions are recorded for each subject, each run containing 42 different trials, equally divided between the 7 classes. Each trial is structured as follows: at second 0, a beep sound starts and a green cross is displayed on a computer screen. The subjects will then focus its gaze on the cross. At second 2, a cue symbol for the required task appeared on the screen. After the cue display, subjects waits for an arbitrary amount of time, and then starts the corresponding movement for the next 3 s. After the movement execution, subjects goes back to the starting neutral position. After the movement, subjects waited in the resting position for a random amount of time, (between 2 and 3 s), before the beginning of the next trial. The data acquisition frequency is 512 Hz, recorded using active electrodes and four 16-channel amplifiers. Reference was placed on the right mastoid, ground on AFz.

### 2.2. Data Epoching and Movement Onset Detection

This work focuses on the decoding of motor intention from EEG signals, therefore movement onset was determined following the approaches originally proposed in Ofner et al. [14]. Moreover, the movement onset detection was further enhanced by implementing a threshold-based algorithm which empirically resulted in more precise and noise-robust motion onset identification (see Appendix A for details). However, in order to not include any movement related data but only motor preparation data in the analysis, the predicted motion onset was visually reviewed for all of the extracted trials. The training epochs have been selected as the part of EEG signals corresponding to one second preceding the detected movement onset. Thus, at the end of the preprocessing stage, our dataset is composed of 420 epochs per subject (for a total of 6300 epochs), equally subdivided into the 7 classes. Finally, we merge the movement classes that involved the motion of the same articulation. The classes “elbow flexion” and “elbow extension” are merged into “elbow”, “wrist pronation” and “wrist supination" into “wrist”, “hand open” and “hand close” into “hand”, thus obtaining four final target classes.

### 2.3. Input Representation and Preprocessing

In order to make our method suitable for real-time application, we decided to perform only a very limited form of preprocessing, that can be efficiently implemented and applied in real-time on the incoming signals. Such preprocessing has been performed before the epoching of the data. We apply a notch filter at 50 Hz to remove power line interference. Then, we apply a 5th-order Butterworth bandpass filter from 0.5 Hz to 60 Hz and raw signals were downsampled from 512 Hz to 128 Hz. In order to fully exploit the implicit spatial information contained in the EEG signals, we arrange the channels at each timestep in a 2-dimensional 10×9 matrix as shown in Table 1. Empty elements of the matrix are set to constant 0. In contrast with other works than can be found in the literature that use only a 1-dimensional spatial arrangement of the channels [25,26], this 2-dimensional arrangement is a more faithful abstraction of the actual deployment of the EEG electrodes on the skull, thus allowing for the complete spatial convolutions of the input signals while avoiding to introduce spurious spatial correlations that may harm the overall performance on the downstream tasks. Since EEG channel names were not available for subject 1, making impossible to arrange the inputs into the desired representation (Table 1), the relative dataset was excluded from the rest of the analysis.

### 2.4. Architecture of the Model

**Figure 2:**
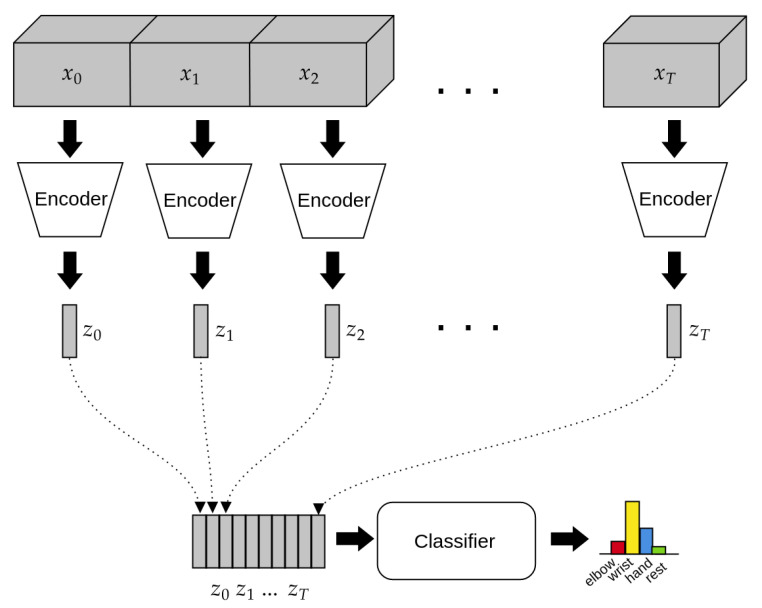
Schematic view of the architecture of the model. Each chunk of input data $x_{i}$ is a sequence of 32 timesteps, with input channels arranged according to Table 1. The Encoder (a 3D convolutional neural network, see Section 2.4.1) processes each input chunk sequentially, producing a series of compressed codes $z_{i}$ as a results. The code sequence is then passed to the Classifier, implemented as a long short-term memory (LSTM) network (see Section 2.4.2), which outputs the final classification at each timestep.

In EEG timeseries, the information resides both in the spatio-temporal localization of patterns and in the frequency components of the signals. Therefore, CNNs seem the most suitable model for this kind of data. In our model, we employ 3-dimensional convolutions in order to process both the spatial an the time dimensions at the same time, thus building more informative representations for the downstream classifier. Such representations are then fed to the recurrent classifier, which employs an RNN to take into account the global context of the data for the final classification step. The model’s architecture, depicted in Figure 2, is composed of two parts: the Encoder and the Classifier. The Encoder’s task is to take in input the preprocessed EEG signals and to produce a compact representation that retains only the information needed for the downstream task. On the other hand, the Classifier takes in input the time series of compact representations produced by the encoder and performs the final classification. A detailed list of the layers composing our model architecture is reported in Table 2.

#### 2.4.1. Encoder

The input representation resulting from the preprocessing procedure described in Section 2.3, albeit able to fully leverage the structure of the raw data, still contains many redundant information that is not actually needed for the final task. First of all, we have spatial redundancy, with many input features set to constant 0, thus not conveying any information about the task. Furthermore, the coarse spatial resolution of EEG electrodes causes the different channels to contain highly correlated signals. We also have temporal redundancy, since in our tasks the bulk of relevant information is contained into the relatively slow temporal evolution of the channels over time rather than in the specific time-local patterns of activation. It is therefore helpful for the final performance to further process the input signals in order to remove those redundancies and to extract only the relevant information. Specifically, the main building block of the encoder are the two 3-dimensional convolutional layers, performing the following computation on the input signals:

(1) $$h\left(N_{i},C_{h_{j}}\right)=b\left(C_{h_{j}}\right)+\sum_{k=0}^{C_{x}-1}W\left(C_{h_{j}},k\right)☆x\left(N_{i},k\right)$$

where *N* is the batch size, $C_{h}$ and $C_{x}$ are respectively the number of input and output channels, *x*, *W* and *b* are the input, weights and bias of the convolutional layer respectively, while ☆ denotes the 3D cross-correlation operator, defined as:

(2) $${\left(k☆x\right)}_{n}=\sum_{m=0}^{N-1}x_{m}k_{(m+n)\%n}$$

where $x\in R^{N}$, $x\in R^{M}$. The 3D convolutions allow the model to take into account, at the same time, both the spatial and the temporal local contexts of the single channels, thus resulting in richer and more informative internal representations during training. The first convolutional layer takes in input a 3D matrix of 32 timestep (so that the total inputs dimension is 32×10×9=2880 input features), applying 16 convolutional channels with convolutions kernel of dimensions (5×2×2) with stride (1×1×1). The output of the convolutions are passed through a ReLU nonlinearity

(3) ReLU(x)=max(0,x)

and a batch normalisation layer [27]: for each input feature of a minibatch *B* $x_{B}^{\left(k\right)}$ the following operations are computed

(4) $$\begin{array}{cc} \mu_{B}^{\left(k\right)} & =\frac{1}{m}\sum_{i=1}^{m}x_{B_{i}}^{\left(k\right)} \end{array}$$

(5) $$\begin{array}{cc} \sigma_{B}^{2(k)} & =\frac{1}{m}\sum_{i=1}^{m}{\left(x_{B_{i}}^{\left(k\right)}-\mu_{B}^{\left(k\right)}\right)}^{2} \end{array}$$

(6) $$\begin{array}{cc} {\hat{x}}_{B}^{\left(k\right)} & =\frac{x_{B}^{\left(k\right)}-\mu_{B}^{\left(k\right)}}{\sqrt{\sigma_{B}^{2(k)}+\epsilon }} \end{array}$$

(7) $$\begin{array}{cc} y_{i} & =\gamma {\hat{x}}_{B}^{\left(k\right)}+\beta \end{array}$$

First, the mean $\mu_{B}^{\left(k\right)}$ and the variance $\sigma_{B}^{2(k)}$ are computed for the current minibatch. Then, the input features are normalized accordingly to the computed values (the additional constant ϵ is added to the denominator for numerical stability). Finally, the normalized input are scaled and shifted by the layer parameters β and γ. Having batch normalization layers in-between convolutional layers helps to mitigate the problem known as internal covariate shifts [27], making the training process more stable. After batch normalization, another 3D convolution is performed on the resulting features, increasing the convolutional channels from 16 to 32 and kernel dimensions (5×2×2), stride (1×1×1). The result of the convolutions is again passed through a ReLU nonlinearity and a batch normalization layer, similarly to the previous layer. Then, a max pooling operation is applied on the output features with a pooling window of size 3×2×2 and stride 3×2×2, in the following way

(8) $$y{\left(N_{i},C_{j}\right)}_{d,h,w}=\max_{k=0,\cdots ,D-1}\max_{m=0,\cdots ,H-1}\max_{n=0,\cdots ,W-1}x{\left(N_{i},C_{j}\right)}_{\mathrm{stride}[0]\times d+k,\mathrm{stride}[1]\times h+m,\mathrm{stride}[2]\times w+n}$$

where *N* is the batch size, *C* is the number of channels, *D*, *H* and *W* are the dimension of the pooling kernel, stride is a vector containing the stride values for each dimension. After the max pooling operation, the representation is flattened into a one-dimensional array of size 576, which is then finally passed through a fully connected layer of ReLU units in order to obtain the 128 features that compose the final representation, followed by a final batch normalization layer. This way, the encoder takes in input the raw data and produces a compact, single-vector representation of the inputs every 32 timesteps (corresponding to 1/4 of a second). This sequence of representations is passed in real-time to the recurrent classifier (see Section 2.4.2).

#### 2.4.2. Classifier

The Classifier is composed of a single Long Short-Term Memory (LSTM) layer [28] with 128 input units and 64 recurrent units. The LSTM layer receives an input $x_{t}$ at time *t* and computes the corresponding output according to the following equations:

(9) $$i_{t}=\sigma (W_{ii}x_{t}+b_{ii}+W_{hi}h_{t-1}+b_{hi})$$

(10) $$f_{t}=\sigma (W_{if}x_{t}+b_{if}+W_{hf}h_{t-1}+b_{hf})$$

(11) $$g_{t}=\tanh(W_{ig}x_{t}+b_{ig}+W_{hg}h_{t-1}+b_{hg})$$

(12) $$o_{t}=\sigma (W_{io}x_{t}+b_{io}+W_{ho}h_{t-1}+b_{ho})$$

(13) $$c_{t}=f_{t}⊙c_{t-i}+i_{t}⊙g_{t}$$

(14) $$h_{t}=o_{t}⊙\tanh\left(c_{t}\right)$$

where $h_{t}$ is the hidden state at time *t*, $c_{t}$ is the cell state at time *t*, $h_{t-1}$ is the hidden state of the layer at time t−1 or the initial hidden state at time 0, $i_{t},f_{t},g_{t},o_{t}$ are respectively the input, forget, cell and output gates. The term σ and tanh denote the sigmoid and the hyperbolic tangent functions, respectively, i.e.,

(15) $$\begin{array}{cc} \sigma (x) & =\frac{1}{1+\exp(-x)} \end{array}$$

(16) $$\begin{array}{cc} \tanh(x) & =\frac{\exp(x)-\exp(-x)}{\exp(x)+\exp(-x)} \end{array}$$

where exp is the exponential function. Finally, ⊙ denotes the Hadamard product. Using an RNN with gating mechanisms allows for the model to construct an internal representation that selectively discards the unimportant information and summarizes the whole sequence up to time *t*, thus making it able to capture the long-term and relatively slow evolution of the input signals instead of the specific local patterns. The network receives at every timestep the compact representations of the encoder and produces an output, which is then passed through a softmax layer

(17) $$\mathrm{Softmax}\left(x_{i}\right)=\frac{\exp x_{i}}{\sum_{j}\exp\left(x_{j}\right)}$$

with four units, to get the final class probabilities (See Figure 2). This way the model yields one classification output every 32 timesteps, corresponding to 0.25 s, making it suitable to be used in real-time tasks.

### 2.5. Training Scheme

The model selection procedure is organised according to a cross-validation scheme, where the training data is divide into 5 folds, 1 of which is kept as the validation set and the other 4 as the training set. The cross-validation is repeated 3 times in order to average out possible extreme results due to the specific random initialisation of the weights. This procedure is repeated for each subject of the dataset, finally yielding 14 models as a result, one for each subject. Training is done via the Adam optimizer [29] for a maximum of 100 epochs. An early stopping strategy is used when the performance on the validation set fail to decrease for 10 consecutive epochs. the model is trained using categorical crossentropy with label smoothing (smoothing parameter 0.2) as a loss function. Label smoothing has the effect to penalize overconfident prediction of the model on specific data samples, making the model less prone to overfitting and more robust to possible noise that might be introduced during the sequence labelling procedure described in Section 2.1. The most important parameters of the model and the optimiser such as learning rate, dimension of the encoder’s representation, dimension of the LSTM hidden state, are chosen according to a grid search strategy, selecting the combination of parameters that yielded the best average performance on the repeated cross-validation procedure described above. Other hyperparameters, such as the batch size and the number of convolutional kernels, have been selected using a trial and error approach. Table 3 shows the grid of hyperparameters that has been used during the model selection procedure. The model is implemented in Python, using the deep learning library of PyTorch. The model has been trained on a NVIDIA Tesla V100 GPU. The total training time for one single subject is of about 12 h.

### 2.6. Comparison with Previous Work

Our final classification accuracy is compared with the previous work by Mammone et al. [24], which focuses on the same dataset used in this work. However, they train a panel of 21 binary classifiers, one for each pair of classes out of the original 7 in the dataset (elbow flection, elbow extension, wrist pronation, wrist supination, hand open, hand close, rest). Hence, the first step needed to ensure a fair comparison is to equalize the number of classes. We achieve this by merging all the classifiers that are related to the same articulation class into a single binary classifier. For example, we merged the “elbow flection vs movement” classifiers with “elbow extension vs movement” classifiers into one single “elbow vs movement” classifier, setting its accuracy as the maximum accuracy among the original classifiers. After the merging step, we obtain 6 different binary classifiers, one for each possible pair of the “elbow”, “wrist”, “hand” and “rest” classes. Still, Mammone et al. [24] do not provide a method for combining the result of these classifications in order to get a final classification of the movement. Therefore, we performed a Monte Carlo estimation of the aggregated accuracy of the binary classifiers. Specifically, we simulated each binary classifier with a Bernoulli random variable, setting the parameter *p* of the Bernoulli distribution according to the accuracy of the specific classifier. We chose the final outcome of the classification following the majority vote strategy, uniformly sampling from the most likely outcomes in case of parity between two or more classes. We performed 10,000 simulations in order to get a reliable estimation of the final aggregated performance. The final accuracy obtained is reported in Table 4.

## 3. Results

### 3.1. Classification Accuracy

**Figure 3:**
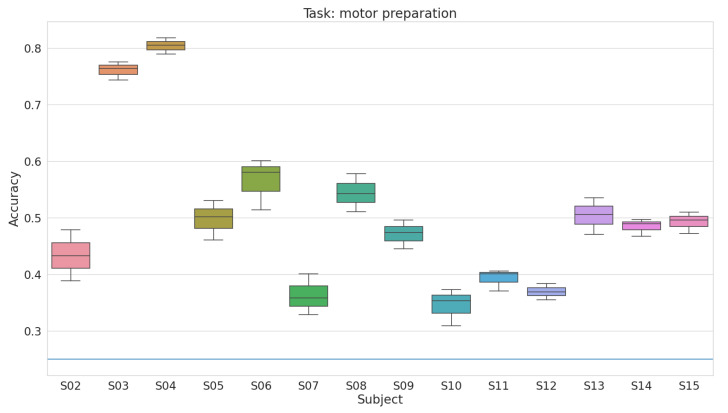
Test accuracy for each of the subjects. The accuracy is averaged over the considered epoch. The results are aggregated across folds. The blue line denotes the accuracy of a random classifier.

In Figure 3 is reported the accuracy of the model on the different subjects of the dataset. The final accuracy has been obtained by feeding to the model the entire epoch, collecting the accuracy of the classifier over time, and then computing the average accuracy over the selected epoch (i.e., the 1 s preceding movement onset). We repeat this process for each fold of the cross validation. In Table 4, the performance of the proposed approach is compared with that by Mammone et al. [24]. The results shows that our approach is well above the accuracy of 25% of the random classifier for all the subjects, and its performance are statistically higher with respect to the ones obtained by the previous work of Mammone et al. [24], despite the much more restrictive setting. We obtain a mean accuracy of 50.79±13.31 and 44.59±10.89 respectively, *t*-test t(13) = 2.21, *p* < 0.05. Normality of data has been checked using Lilliefors test, and in both cases the null hypothesis was not rejected.

### 3.2. Performance over Time

**Figure 4:**
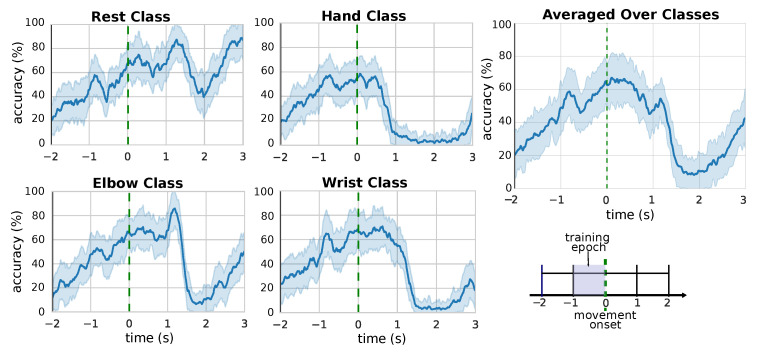
Accuracy of the model, at each timestep, in the time segment around the detected movement onset, for the different movement classes. The trials have all been synchronized at the actual movement onset (t=0), denoted by the green dashed line (see Section 2.1). The figure in the bottom-right corner describes the structure of the trials (see Section 2.1) at *t* In the experiments, the model is trained only using the 1-second segment preceding the movement onset.

Since we aim at using our approach in real time, we are also interested in the temporal evolution of the model’s performance, not only in its aggregated statistics. For this reason, we inspect the accuracy of our model during all the duration of the epoch of interest. In Figure 4 is reported the time-wise accuracy over the selected epoch. The black solid line denotes the moment when the green cross is displayed on screen (see Section 2.1), the green dashed line denotes the moment the subject actually start to perform the corresponding movement. It is possible to see that the classifications become more and more correct as the time increase, eventually reaching maximum accuracy shortly after the actual start of the movement, at about t=−0.25. This is in line with the model expected behaviour: as more data becomes available, the model processes it and stores the important information in the hidden state of the LSTM, thus building an increasingly accurate estimation of the movement intention. Interestingly, the plots show that the performance stays high even shorty after movement onset (i.e., the green line), a sign that the model is able to generalize to unseen data during the inference phase (note that the model is trained only on the 1-second segment preceding the green line). After the movement execution, the performance quickly drops, and the model gets ready for the classification of another movement. The time-wise accuracy for the single movement class reveals a similar behaviour for all types of movements, meaning that the performance of the model is not skewed towards a particular class. In the case of the “rest” class, we do not observe a rapid drop of performance after the movement execution. This can be explained by the fact that, for the “rest” class, there are less significant differences between the performance of the “rest action” and the idle time before and after the action itself.

## 4. Discussion

In this work, we implemented a novel neural network architecture for the real-time detection of movement intentions. The model works directly with raw EEG signals and leverages a topology-aware input representation to extract both spatial and temporal dependencies of the inputs at the same time. The architecture of a convolutional neural network (CNN) allows us to process not only the information contained in a single channel, but also the information stored implicitly into the spatial relations between a channel and its neighbours. Due to the relatively coarse spatial resolution of EEGs, neighbouring channels have the tendency to capture signals that are highly correlated with each other, as often it is more useful to consider the co-evolution of groups of channels rather than the evolution of each individual channel. This makes this kind of data a perfect fit for CNNs.

The empirical analysis shows that our model yields superior performance with respect to previous work on the same dataset [24], despite being trained in a much more restrictive setting in order to satisfy the requirements of real-time applications. Firstly, we perform an efficient one-shot multiclass classification, classifying all the four movements at the same time, instead of using a panel of binary classifiers, thus avoiding the need of implementing an aggregation strategy that introduces additional error. In fact, it has to be considered that, when using the One-Against-One approach (i.e., generating $\frac{k(k-1)}{2}$ binary classifiers with k classes), the final classification of a specific binary classifier is meaningless when the data sample does not belong to neither classes of that classifier. Hence, the final classification may be damaged if most of these irrelevant classifiers make erroneous predictions [30]. Secondly, our model is able to perform classification at each timestep of the epoch, converging on average to the correct class after about 0.25 s before the movement onset. This allows our model to process a continuous stream of data in real-time, without the need of identifying the start and the end of the epoch before feeding it to the model, making it suited for its deployment in real-world applications. On the other hand, the models in [24] perform a single classification at the end of the epoch, at the movement onset, after the whole training epoch is fed as input.

Our model employs a simple and lightweight form of preprocessing, only consisting in a combination of band-pass and notch filters, since our topology-preserving representation removes the need of using complex source-space reconstructed representations as in [24]. Interestingly, the model seems able to naturally generalise to unseen data, as the classification performance keeps staying high not only before, but even during the movement itself, despite the model was never being trained on such data.

The ability to perform reliable movement intention recognition is crucial for a wide range of real-time BCI applications, as it allows to mask the delay that naturally occurs between the recognition of the user’s movement and the actuation of the corresponding action in the brain-controlled device. Moreover, in our previous work [31] we demonstrated the suitability of an online application of the proposed convolutional encoder architecture within the ROS framework. In this paper we showed that deep learning models can be effectively used to solve this kind of tasks, while not relying on any particular form of intensive preprocessing methods.

In the future, we plan to further refine the input representations, implementing the exact spatial location of the EEG channels via e.g., a point-cloud representation of the input signals. This representation can be exploited by a deep graph network [32] to produce an even more informative representation for the downstream classifier. It will be interesting to experiment with different sensors’ configurations, especially with a reduced sensors set. We also plan to perform an extensive electrophysiological analysis of the information retained by the encoder module in order to understand which part of the signals are the most relevant for the final classification.

## Figures and Tables

**Table 1:** Spatial arrangement of input channels at each timestep. The blank entries are filled with 0 s.

		F3	F1	Fz	F2	F4		
	FFC5	FFC3	FFC1		FFC2	FFC4	FFC6	
	FC5	FC3	FC1	FCz	FC2	FC4	FC6	
FTT7	FCC5	FCC3	FCC1		FCC2	FCC4	FCC6	FTT8
	C5	C3	C1	Cz	C2	C4	C6	
TTP7	CCP5	CCP3	CCP1		CCP2	CCP4	CCP6	TTP8
	CP5	CP3	CP1	CPz	CP2	CP4	CP6	
	CPP5	CPP3	CPP1		CPP2	CPP4	CPP6	
		P3	P1	Pz	P2	P4		
			PPO1		PPO2			

**Table 2:** List of layers that compose each module of the model. Layers name and parameters.

Module	Layers
Encoder	Conv3d (1, 16, kernel_size = (5, 2, 2), stride = (1, 1, 1))
	ReLU()
	BatchNorm3d (16, eps = $10^{-5}$, momentum = 0.1, affine = True, track_running_stats = True)
	Conv3d (16, 32, kernel_size = (5, 1, 1), stride = (1, 1, 1))
	ReLU()
	BatchNorm3d (32, eps = $10^{-5}$, momentum = 0.1, affine = True, track_running_stats = True)
	MaxPool3d (kerne_size = (3, 2, 2), stride = (3, 2, 2), padding = 0, dilation = 1, ceil_mode = False)
	Linear (in_features = 576, out_features = 128, bias = True)
	ReLU()
	BatchNorm1d (128, eps = $10^{-5}$, momentum = 0.1, affine = True, track_running_stats = True)
Classifier	LSTM (input_size=128, hidden_size = 64, bidirectional = False, dropout = 0)
	Linear (in_features = 64, out_features = 4, bias = True)
	Softmax (n_classes = 4)

**Table 3:** Grid of hyperparameters taken into consideration during cross-validation.

Name	Description	Values
batch_size	Number of neurons of the encoder’s output.	1024
z_dim	Number of neurons of the encoder’s output.	[512, 256, 128]
lstm_hidden_size	Number of neurons of LSTM’s hidden state.	[32, 64, 128]
conv1_channels	Number of channels of first convolutional layer.	[8, 16, 32]
conv2_channels	Number of channels of second convolutional layer.	[32, 64, 128]
lstm_depth	Number of LSTM layers.	1
conv_depth	Number of convolutional layers.	2
η	ADAM initial learning rate.	[0.001, $10^{-4}$, $10^{-5}$]
$\beta_{1}$	ADAM $\beta_{1}$ parameter.	0.9
$\beta_{2}$	ADAM $\beta_{2}$ parameter.	0.999
lstm_dropout	Percentage of dropped units in the LSTM layers.	0
smoothing	Amount of label smoothing.	[0, 0.2, 0.4]
L2_penalty	Amount of L2 regularization during training (weight decay).	0

**Table 4:** Final accuracy of the model for each subjects. Bold entries represent the best accuracy for that subject. Higher is better.

Subject	Our Work	Previous Work [24]
Subject 02	**42.58 ± 1.56**	39.09
Subject 03	**76.72 ± 0.51**	57.86
Subject 04	**81.44 ± 0.35**	68.69
Subject 05	**50.05 ± 1.74**	38.62
Subject 06	**58.22 ± 2.04**	36.94
Subject 07	**36.39 ± 1.50**	28.02
Subject 08	**54.82 ± 1.66**	41.12
Subject 09	**47.55 ± 0.89**	45.72
Subject 10	36.01 ± 1.34	**58.17**
Subject 11	**40.05 ± 0.29**	37.62
Subject 12	**37.50 ± 0.26**	35.48
Subject 13	**50.98 ± 1.31**	47.37
Subject 14	49.22 ± 0.17	**49.85**
Subject 15	**49.56 ± 0.45**	39.80
Average	**50.79 ± 13.82**	44.59 ± 10.89

## Data Availability

All data used in this study is available at http://bnci-horizon-2020.eu/database/data-sets (accessed on 22 December 2020).

## Appendix A. Enhanced Movement Onset Detection Heuristic

We developed the following heuristic to identify the motion onset: we start by considering the signals coming from the accelerometers installed in both the exoskeleton and the glove. We first compute their baseline values by considering their median values in the half-second preceding the cue display (i.e., the median values in the interval t=[1.5,2] s). We compute the medians $\mu_{i}$ of each signal separately, where *i* is the index of the specific channel. For each channel *i* we set a “motion threshold” $\theta_{i}=\left|\frac{1}{10}\mu_{i}\right|$. Intuitively, this means that we detect a movement for a channel if the signal deviates from its median by more than 10%. After cue display, at time t=2 s, we monitor the evolution of the motions channels. At each timestep, we compute for each signal the absolute deviation from its median, and compare it to the threshold

(A1) $$\left|x_{i,t}-\mu_{i}\right|>\theta_{i}$$

where $x_{i,t}$ is the value of channel *i* at time *t*, $\mu_{i}$ and $\theta_{i}$ are respectively the median and the threshold for the *i*-th channel. To make this procedure more robust to the noise of a single channel, we consider a particular timestep *t* to be a “motion timestep” if and only if at least three channels exceed their motion threshold as described in Equation (A1). Additionally, we further avoid false positive by introducing a patience parameter, set to 20 timestep. Thus, we finally decree the start of movement only if we detect a more than 20 “motion timesteps” in a row. The “motion onset” for the rest “class” was taken as in [14]. This procedure was visually validated on the dataset. We empirically found that our methods results in more precise and noise robust motion onset identification than the one described in [14].
